# Development and Validation of an UPLC-MS/MS Method for Pharmacokinetic Comparison of Five Alkaloids from JinQi Jiangtang Tablets and Its Monarch Drug Coptidis Rhizoma

**DOI:** 10.3390/pharmaceutics10010004

**Published:** 2017-12-29

**Authors:** Lili Sun, Feifei Ding, Guangjiao You, Han Liu, Meng Wang, Xiaoliang Ren, Yanru Deng

**Affiliations:** 1 School of Chinese Materia Medica, Tianjin University of Traditional Chinese Medicine, Tianjin 300193, China; 18322050489@163.com (L.S.); vampiredff@163.com (F.D.); 15022169561@163.com (G.Y.); liuhanhebei@163.com (H.L.); 2 Tianjin State Key Laboratory of Modern Chinese Medicine, Tianjin University of Traditional Chinese Medicine, Tianjin 300193, China; mengwangr@163.com

**Keywords:** JinQi Jiangtang tablets, Coptidis Rhizoma, pharmacokinetics, UPLC-MS/MS

## Abstract

JinQi Jiangtang (JQJT) tablets, a Chinese patent medicine approved by the State Food and Drug Administration, are composed of Coptidis Rhizoma, Astragali Radix, and Lonicerae Japonicae Flos, and have a significant effect on diabetes. Coptidis Rhizoma is monarch drug in the prescription. The aim of the present study was to investigate and compare the pharmacokinetics of multiple ingredients from JQJT tablets and Coptidis Rhizoma extract (CRE) following oral administration in rats. Five alkaloids: coptisine chloride, epiberberine chloride, berberine chloride, jatrorrhizine chloride, and palmatine chloride, were simultaneously determined in rat plasma using established and validated ultra-high performance liquid chromatography mass spectrometry (UPLC-MS/MS). Significant pharmacokinetic differences were observed for the five alkaloids after a single administration of CRE and JQJT tablets. Compared with CRE, the C_max_ values of palmatine chloride and jatrorrhizine chloride were decreased significantly, the AUC_0–*t*_ values of four alkaloids (all except jatrorrhizine chloride) were notably decreased, and the mean residence times of all five alkaloids were significantly decreased after administration of JQJT tablets. The results indicated that the absorption characteristics of the five alkaloids from Coptidis Rhizoma would be influenced by the compatibility of Astragali Radix or Lonicerae Japonicae Flos from JQJT tablets, such that absorption was inhibited and elimination was accelerated. In conclusion, the developed strategy was suitable for the comparison of five alkaloids from JinQi Jiangtang tablets and its monarch drug, which could be valuable for compatibility studies of traditional Chinese medicines.

## 1. Introduction

Recently, Traditional Chinese Medicine (TCM) has attracted increasing attention, not only as single herbs but also including Chinese medicinal compounds, Chinese patent medicines, and acupuncture [1,2,3]. JinQi Jiangtang (JQJT) tablets, composed of Coptidis Rhizoma, Astragali Radix, and Lonicerae Japonicae Flos, are recorded in the Pharmacopeia of the People’s Republic of China (Chinese Pharmacopoeia Committee, 2015), and originated in Essential Prescriptions Worth a Thousand Pieces of Gold (Qian Jin Yao Fang, in Chinese) written by Sun Simiao during the Tang dynasty [4,5]. The JQJT tablets, a Chinese patent medicine, have a significant effect on diabetes and are approved by the State Food and Drug Administration [6,7]. The various components of JQJT tablets include alkaloids, organic acids, and flavonoids, which contribute to a variety of pharmacological properties, such as hypoglycemic and hypolipidemic effects, free radical scavenging activity, and immune regulation [8,9].

Coptidis Rhizoma (Huanglian in Chinese), derived from *Astragalus membranaceus* (Fisch.) Bge. var. *mongholicus* (Bge.) Hsiao or *Astragalus membranaceus* (Fisch.) Bge., is the monarch drug in JQJT tablets. It is a Chinese medicinal herb that has various pharmacological properties, including anti-inflammatory, antioxidant, anti-cancer, immune regulation, and antimicrobial activities, and has been widely used to treat cardiovascular diseases, gastro enteric disorders, and cancer, amongst others [10,11]. In addition, it has been suggested that Coptidis Rhizoma has potential as a cardiovascular protective agent and a cancer chemopreventive agent [10,12]. The active constituents of Coptidis Rhizoma comprise a large number of alkaloids, the major ones being berberine, jatrorrhizine, palmatine, epiberberine, and coptisine [13,14]. It is well known that traditional Chinese medicine is a complex system, and combinations can make the prescriptions more suitable for clinical use and produce herb-herb interactions that improve pharmacological properties. Pharmacokinetic study of multiple ingredients could be useful to elucidate herb-herb interactions. Moreover, there is no reported analytical method for the simultaneous detection of five alkaloids and the comparison of their pharmacokinetics following administration of JQJT tablets or their principal drug component (Coptidis Rhizoma).

**Figure 1:**
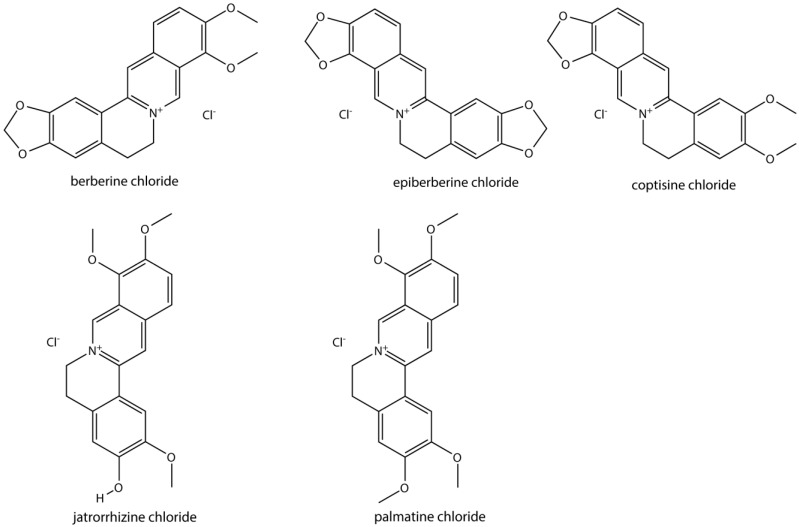
Structures of the five alkaloids.

In this study, five alkaloids—berberine, epiberberine, coptisine, jatrorrhizine, and palmitine (Figure 1)—were detected in rat plasma by ultra-high performance liquid chromatography-triple quadrupole tandem mass spectrometry (UPLC-MS/MS). Pharmacokinetics of the five alkaloids in vivo were compared following oral administration of JQJT tablets or Coptidis Rhizoma extract.

## 2. Experimental

### 2.1. Chemicals and Reagents

JinQi Jiangtang tablets and Coptidis Rhizoma were supplied by Tianjin Zhongxin Pharmaceutical Group Corp. Ltd., Longshunrong Pharmaceutical Factory (Tianjin, China). Berberine chloride, jatrorrhizine chloride, and palmatine chloride were obtained from the National Institute for the Control of Pharmaceutical and Biological Products (Beijing, China). Epiberberine chloride and coptisine chloride were purchased from Chengdu Mansite Biological Technology Co., Ltd. (Chengdu, China) Methanol, formic acid, and acetonitrile of HPLC grade were purchased from Fisher Scientific (Waltham, MA, USA), MREDA Technology Inc. (Dallas, TX, USA), and Fisher Scientific, respectively. Water was purified on a Milli-Q system (Millipore, Bedford, MA, USA). Heparin was purchased from Tianjin Biochemical Pharmaceutical Co., Ltd. (Tianjin, China). Other reagents were of analytical grade and obtained from Concord Technology Co., Ltd. (Tianjin, China).

### 2.2. Animals

Eight-week-old male Wistar rats (220 ± 20 g) were supplied by Tianjin Shanchuanhong Laboratory Animal Technology Co., Ltd. (Tianjin, China) (SCXK 2009(jin)-0001). The animals were raised in a suitable environment with controlled temperature (22 ± 2 °C) and relative humidity (50% ± 10%) for one week before experimentation to allow them to acclimate. The animal experiment abided by the National Institutes of Health Guide for Care and Use of Laboratory Animals.

### 2.3. UPLC-MS/MS Conditions

Ultra-high performance liquid chromatography mass spectrometry (UPLC-MS/MS) analysis was carried out on a Waters Acquity UPLC-Quattro Premier XE tandem mass spectrometer (Waters Co., Milford, MA, USA) with an Acquity UPLC BEH Shield RP_18_ column (1.7 μm, 2.1 × 100 mm). Mobile phases A (0.1% formic acid in water) and B (acetonitrile) were used for gradient elution as follows: 0–1 min (10–20% B), 1–3 min (20–22.5% B), 3–3.5 min (22.5–32.5% B), 3.5–4.5 min (32.5–45% B), 4.5–5 min (45–90% B), 5–5.51 min (90–10% B), 5.51–8 min (10% B). The flow rate was 0.3 mL/min. The column temperature was kept at 25 °C, and the injected volume was 5 μL.

Mass analysis was performed on a Quattro Premier XE triple quadrupole mass spectrometer with an electrospray ionization (ESI) source in positive mode. The MS parameters were as follows: source temperature of 120 °C, capillary voltage of 3.2 kV, desolvation temperature of 350 °C, desolvation gas flow rate of 600 L/h, cone gas flow rate of 50 L/h, and dwell time of 0.08 s. Multiple reaction monitoring (MRM) was used to conduct the quantitative analysis, and the optimized MS parameters are shown in Table 1.

### 2.4. Preparation of Stock Solutions, Calibration Curve, Quality Control Samples, and Biological Plasma Samples

Reference standards of coptisine chloride, epiberberine chloride, berberine chloride, jatrorrhizine chloride, palmatine chloride, and propranolol (IS) were separately prepared in DMSO (dimethyl sulfoxide) at a concentration of 1.0 mg/mL. The stock solutions were diluted with 50% aqueous methanol to yield mixed standard solutions at appropriate concentrations.

Calibration curve samples were prepared by adding 5 μL of standard solutions and 150 μL of internal standard (IS) solution to 50 μL blank plasma, vortex mixing for 1 min, and centrifugation at 18,000× *g* for 10 min. Eight different concentrations of calibration curve samples were used: 0.2, 0.5, 1, 2, 5, 10, 20, 50, and 100 ng/mL.

Quality control (QC) samples were prepared by the same procedure to achieve low (1 ng/mL), medium (5 ng/mL), and high (50 ng/mL) concentration levels. All of the solutions were stored in a freezer at −80 °C.

The plasma sample (50 μL) was mixed with 150 μL of IS by vortex mixing for 1 min. After centrifugation at 14,000 rpm for 10 min, the supernatant was used for UPLC-MS/MS analysis.

### 2.5. Preparation of Coptidis Rhizoma Extract

Coptidis Rhizoma was extracted with 70% aqueous ethanol. The extract was concentrated, purified using D101 macroporous adsorption resin, and then evaporated to obtain the dry extract. The total alkaloid contents of JQJT tablets and CRE were determined by UV spectrophotometry at 354 nm. The total alkaloid concentration in the high dose of JinQi Jiangtang tablets was calculated to achieve the same dosage as that of the Coptidis Rhizoma extract. The equivalent doses were 0.4536 g/200 g for JQJT tablets and 0.0650 g/200 g for CRE. The JQJT tablets and CRE were finely powdered and suspended in 0.5% hydroxyl propyl methyl cellulose.

### 2.6. Method Validation

The validation was conducted according to bioanalytical method validation guidance, including the determination of specificity, calibration curve, sensitivity, precision, accuracy, recovery, matrix effect, and stability [15,16,17,18].

The specificity was evaluated by analysis of blank plasma samples from six different rats. Endogenous substances and other co-eluting analytes in blank plasma were observed to identify potential chromatographic interference.

The ratio of analyte peak area to that of IS (Y) versus the concentration of standard (X) was used to establish calibration curves by weighted regression (1/X^2^) least squares regression analysis. The lower limit of quantification (LLOQ), the lowest concentration on the calibration curve, was evaluated via precision (relative standard deviation, RSD ≤ 20%) and accuracy (relative error, RE ≤ 20%) to describe the sensitivity of the method.

Intra- and inter-day precision and accuracy were assessed using QC samples at three concentration levels on the same day and three consecutive days, respectively. The acceptance criteria for precision and accuracy were that the RSD did not exceed 15% and that the RE was within ±15%.

Extraction recoveries were determined by comparing the mean peak areas of normal analytes at three QC concentrations with those achieved from spike-after-extraction. Matrix effects were evaluated by comparing peak areas ratios obtained for analytes dissolved in blank matrix extract versus those in pure standard solutions at the equivalent concentration level. The acceptable level of accuracy should be in the range of 85% to 115%, and the RSD values should be less than or equal to 15%.

QC samples at three different concentration levels were used to determine the stability of analytes in plasmas samples under different conditions, including the short-term stability of each QC sample at room temperature for 2 h, long-term stability at 4 °C for 24 h due to the temperature of samples in autosampler, and freeze–thaw stability after three freeze-thaw cycles (−80 °C to room temperature).

### 2.7. Pharmacokinetic Study

Rats were randomly assigned to two groups that were dosed with JQJT tablets or CRE (*n* = 6). All of the rats were forbidden from feeding, but had access to water, for 12 h before the experiment. Rats were dosed with JQJT tablets (0.4536 g/200 g) or CRE (0.0650 g/200 g). Blood samples were collected from each rat at 5, 10, 20, 30, 45, 60, 90, 120, 150, 180, 240, 360, 480, 600, 720, and 1440 min via the retro-orbital plexus and placed into heparinized tubes. After centrifugation at 6000 rpm for 15 min, the obtained plasma samples were stored at −80 °C until UPLC-MS/MS analysis. DAS (Drug And Statistics) version 1.0 was used to calculate the pharmacokinetic parameters. Additionally, the *t*-test was used to compare the differences of pharmacokinetic parameters between the JQJT tablets group and the CRE group using IBM SPSS Statistics 19.0 software (New York, NY, USA, 2011).

## 3. Results and Discussion

### 3.1. Method Validation

#### 3.1.1. Specificity

**Figure 2:**
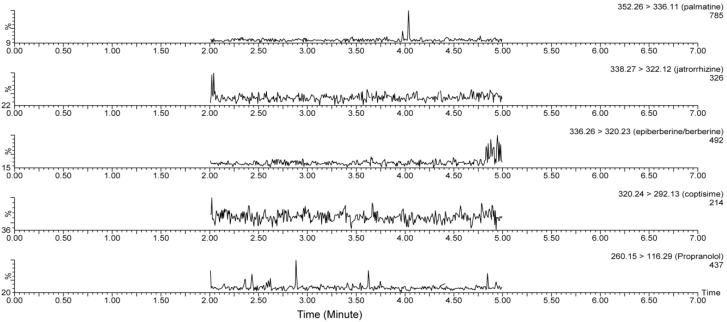
Representative MRM (Multiple reaction monitoring) chromatograms of palmatine, jatrorrhizine, epiberberine, berberine, coptisine, and IS in blank plasma. No endogenous interference was observed in blank plasma.

**Figure 3:**
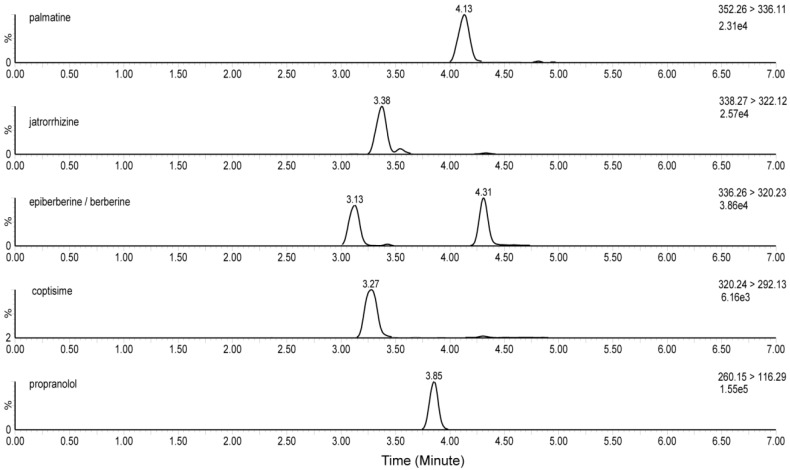
Representative MRM chromatograms of palmatine, jatrorrhizine, epiberberine, berberine, coptisine, and IS in plasma collected 2 h after oral administration of JinQi Jiangtang (JQJT).

As shown in Figure 2 and Figure 3, the specificity of the proposed method was verified. No endogenous interference peak was found at the retention times of the five alkaloids or IS in plasma (Figure 2). Retention times for palmatine chloride, jatrorrhizine chloride, epiberberine chloride, berberine chloride, coptisine chloride, and IS were 4.14, 3.37, 4.31, 3.13, 3.27, and 3.85 min, respectively (Figure 3). The IS and the five alkaloids were simultaneously detected in the same chromatographic run and good separation was achieved.

#### 3.1.2. Calibration Curves and Sensitivity

Calibration curve equations, correlation coefficients (r), and linear ranges for five alkaloids in plasma are presented in Table 2, showing good linearity. The LLOQs (lower limits of quantification) of the analytes were 0.2 ng/mL with an RSD of less than 20% and an RE within ±20%, suggesting the high sensitivity of the proposed UPLC-MS/MS method.

#### 3.1.3. Accuracy and Precision

The results for intra- and inter-day accuracy and precision are shown in Table 3. The intra- and inter-day accuracy (RE) varied from −8.5% to 8.00% and from −6.70% to 7.13%, and the precision (RSD) was less than 10.13% and 6.19%, respectively. Satisfactory accuracy and precision were achieved for the quantification of the five alkaloids in rat plasma.

#### 3.1.4. Extraction Recovery and Matrix Effect

The extraction recoveries of the five alkaloids at three QC concentration levels were 97.7–103.0% for palmatine chloride, 96.8–102.1% for jatrorrhizine chloride, 100.6–106.5% for epiberberine chloride, 94.8–106.2% for berberine chloride, and 97.3–100.2% for coptisine chloride. The results indicated acceptable recovery for the five analytes. The matrix effects for the five alkaloids were in the range of 96.3–108.8%.

#### 3.1.5. Stability

The stability of the five analytes during short-term storage at room temperature for 2 h, long-term storage at 4 °C for 24 h, and three freeze-thaw cycles are shown in Table 4. The RSD of IS areas were in the range of 1.54–4.13%. Moreover, the RSD of resident times obtained from the five alkaloids and IS varied from 0.22–0.45%.

### 3.2. Pharmacokinetic Study

**Figure 4:**
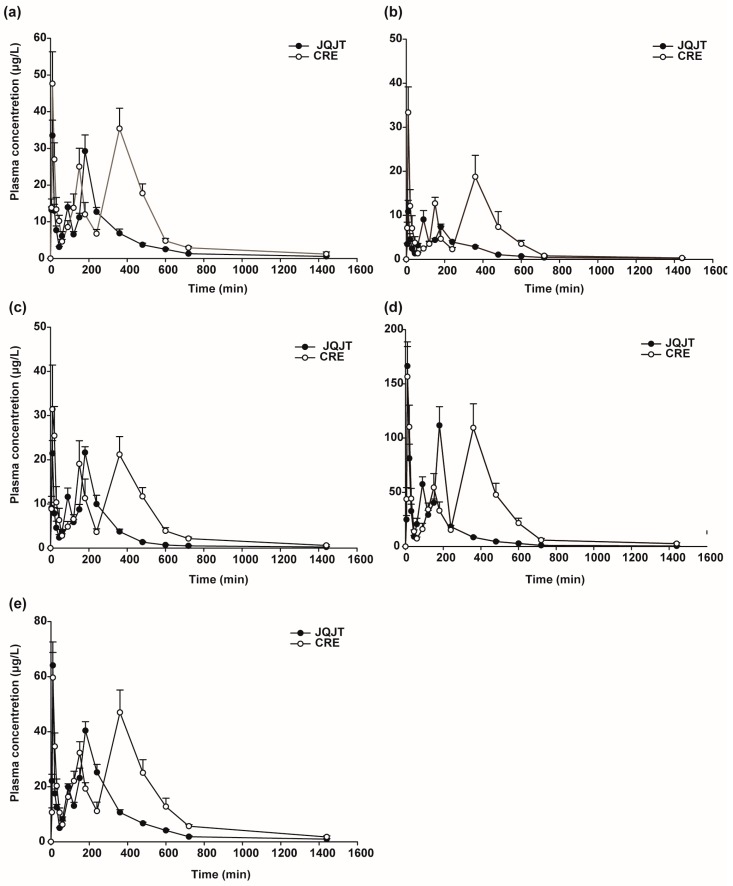
Mean plasma concentration-time profiles of palmatine chloride (**a**), jatrorrhizine chloride (**b**), epiberberine chloride (**c**), berberine chloride (**d**), and coptisine chloride (**e**) in rat plasma after oral administration of JQJT tablets and CRE. The error bars represent the standard deviation obtained from six replicates.

Pharmacokinetics of the five alkaloids following oral administration of JQJT tablets and CRE at equivalent doses of total alkaloids were successfully characterized by the proposed and validated UPLC-MS/MS method. Figure 4 illustrates the mean plasma concentration-time profiles of palmatine chloride, jatrorrhizine chloride, epiberberine chloride, berberine chloride, and coptisine chloride obtained after oral administration of JQJT tablets and CRE. The pharmacokinetics of the five alkaloids fitted a non-compartmental model according to the Akaike information criterion, and the parameters are presented in Table 5, including maximum concentration (C_max_), area under the concentration-time curve (AUC), mean residence time (MTR), and the variance of residence time (VRT). Additionally, mean ± SD was applied to express the results, and the *t*-test was used to compare the differences of pharmacokinetic parameters between JQJT tablets group and CRE group.

As shown in Figure 4, multiple peaks were found in the mean plasma concentration-time profiles of the five alkaloids. This is possibly due to enterohepatic circulation, during which the absorbed alkaloids are excreted via bile into the intestinal tract and then re-absorbed [19,20,21,22]. Compared to oral administration of CRE, the C_max_ values for palmatine chloride, jatrorrhizine chloride, and epiberberine chloride were decreased significantly; the AUC_0–*tn*_ values for palmatine chloride, epiberberine chloride, berberine chloride, and coptisine chloride were significant decreased; the AUC_0–*tn*_ for jatrorrhizine chloride was significantly increased; and the mean residence time (MRT) for all five alkaloids was decreased after oral administration of JQJT tablets. The results revealed that the compatibility of Astragali Radix or Lonicerae Japonicae Flos in JQJT tablets influenced the absorption characteristics of the five alkaloids from Coptidis Rhizoma, such that absorption was inhibited and elimination was accelerated. The chlorogenic acid from Lonicerae Flos is the known potential inhibitor, which has been validated in Reference [19], although other potential inhibitors require further study.

## 4. Conclusions

An UPLC-MS/MS method was established and validated to explore and compare the pharmacokinetics of five alkaloids in rat plasma after oral administration of JinQi Jiangtang tablets and Coptidis Rhizoma extract. Compared with Coptidis Rhizoma extract, the absorption of five alkaloids from JinQi Jiangtang tablets was inhibited and their elimination was accelerated. Excluding the effect of formulation, compatibility with the other herbs (Astragali Radix, and Lonicerae Japonicae Flos) may be the significant factor influencing the absorption characteristics of the five alkaloids from Coptidis Rhizoma in JQJT tablets. For example, various organic acids present in Lonicerae Japonicae Flos and polysaccharides in Astragali Radix could inhibit the absorption of alkaloids [19]. This study provides useful information on the pharmacokinetics of JQJT tablets and the compatibility of Coptidis Rhizoma with two other herbs present in JQJT tablets. However, further in-depth research is required to elucidate the ambiguous mechanism of the inhibited absorption and accelerated elimination. In conclusion, the developed strategy was sensitive, accurate, and suitable for the comparison of five alkaloids from JinQi Jiangtang tablets and its monarch drug, which could be valuable for the compatibility study of traditional Chinese medicines.

## Figures and Tables

**Table 1:** The optimized mass spectrometry (MS) parameters of the five alkaloids and internal standard (IS).

Compound Name	Parent (*m*/*z*)	Daughter (*m*/*z*)	Cone (V)	Collision (eV)
Propranolol (IS)	260.15	116.29	35	18
Coptisime chloride	320.24	292.13	44	29
Epiberberine chloride	336.26	320.23	47	31
Berberine chloride	336.26	320.23	47	31
Jatrorrhizine chloride	338.27	322.12	38	29
Palmatine chloride	352.26	336.11	36	30

**Table 2:** Calibration curve parameters for the five alkaloids in plasma (*n* = 6).

Compound	Calibration Curves	r ^a^	Linear Ranges (ng/mL)
Palmatine chloride	Y = 0.0381X + 0.0034	0.9981	0.2–100
Jatrorrhizine chloride	Y = 0.0341X + 0.0034	0.9987	0.2–100
Epiberberine chloride	Y = 0.0534X + 0.0053	0.9978	0.2–100
Berberine chloride	Y = 0.0581X + 0.00052	0.9985	0.2–100
Coptisime chloride	Y = 0.0108X − 0.0013	0.9976	0.2–100

^a^ r represents the correlation coefficient.

**Table 3:** Accuracy and precision for the five alkaloids in plasma (*n* = 6); RE: relative error; RSD: relative standard deviation.

Analyte	Concentration (ng/mL)	Intra-Day		Inter-Day	
		Accurary (RE %)	Precision (RSD %)	Accurary (RE %)	Precision (RSD %)
Palmatine chloride	1	−3.33	5.97	6.70	5.41
	5	1.33	10.13	2.84	2.00
	50	1.60	2.98	2.50	3.56
Jatrorrhizine chloride	1	−4.00	5.51	−3.30	5.97
	5	4.67	2.21	−1.30	2.34
	50	1.27	1.46	2.70	1.66
Epiberberine chloride	1	−8.50	2.84	−6.70	6.19
	5	8.00	1.85	4.00	3.85
	50	−0.53	0.81	3.70	5.03
Berberine chloride	1	3.33	5.59	−6.70	6.19
	5	3.33	2.96	4.00	3.33
	50	−2.07	1.39	3.10	5.83
Coptisime chloride	1	−4.80	4.74	−6.67	6.19
	5	2.00	8.55	−1.33	2.34
	50	2.80	7.42	7.13	0.96

**Table 4:** The stability of five analytes in plasma at three quality control (QC) concentration levels.

Analyte	Concentration	Room temperature, 2 h		4 °C, 24 h		Three Freeze-Thaw Cycles	
		RSD %	RE %	RSD %	RE %	RSD %	RE %
Palmatine chloride	0.5	5.49	0.33	5.62	−1.00	9.08	−0.67
	5.0	5.19	2.00	0.011	6.67	4.11	1.33
	80.0	3.81	1.67	4.11	0.83	2.27	0.04
Jatrorrhizine chloride	0.5	5.88	−0.33	2.98	2.67	7.63	−1.00
	5.0	2.44	−5.33	3.08	−0.67	4.11	1.33
	80.0	2.79	5.25	4.23	3.17	1.17	2.75
Epiberberine chloride	0.5	6.65	2.33	1.96	2.00	6.98	0.67
	5.0	3.03	0.67	6.26	2.67	6.07	0.67
	80.0	5.67	1.46	4.75	−1.96	2.70	0.25
Berberine chloride	0.5	8.19	−1.33	13.97	−4.67	1.96	2.00
	5.0	1.14	1.33	2.96	3.33	5.00	0.67
	80.0	3.74	3.96	5.51	−1.75	2.43	1.13
Coptisime chloride	0.5	5.88	−0.33	4.44	1.67	6.19	−1.33
	5.0	3.14	−2.67	3.90	6.67	8.14	−0.67
	80.0	1.68	7.50	3.64	1.25	3.24	6.04

**Table 5:** The pharmacokinetic parameters of the five alkaloids from JQJT and Coptidis Rhizoma extract (CRE); concentration-time curve (AUC), mean residence time (MTR); variance of residence time (VRT).

Analyte	Group	C_max_ (ug/L)	AUC_0–*tn*_ (ug/L h)	MRT_0–*tn*_ (h)	VRT_0–*tn*_ (h^2^)
Palmatine chloride	CRE	47.65 ± 8.69	204.86 ± 12.17	6.60 ± 0.50	21.23 ± 5.55
	JQJT	35.1 ± 2.96 *	107.51 ± 6.32 **	5.20 ± 0.26 **	21.87 ± 3.20
Jatrorrhizine chloride	CRE	33.35 ± 5.82	96.58 ± 21.69	6.14 ± 0.30	15.45 ± 1.26
	JQJT	11.35 ± 2.48 **	279.70 ± 83.40 **	5.08 ± 0.42 **	24.55 ± 5.42 **
Epiberberine chloride	CRE	32.60 ± 8.77	125.03 ± 12.84	6.48 ± 0.29	19.61 ± 2.20
	JQJT	22.75 ± 2.15	68.58 ± 5.48 **	4.31 ± 0.19 **	16.09 ± 3.01
Berberine chloride	CRE	156.55 ± 27.85	571.59 ± 44.41	6.46 ± 0.18	18.44 ± 2.84
	JQJT	166.40 ± 22.36	269.36 ± 13.91 **	3.28 ± 0.12 **	10.43 ± 2.19 **
Coptisime chloride	CRE	60.85 ± 7.34	309.59 ± 27.06	6.94 ± 0.35	21.51 ± 3.40
	JQJT	64.10 ± 8.50	177.50 ± 9.83 **	5.179 ± 0.18 **	21.78 ± 2.54

* *p* < 0.05; ** *p* < 0.01.
